# Visual attention is not deployed at the endpoint of averaging saccades

**DOI:** 10.1371/journal.pbio.2006548

**Published:** 2018-06-25

**Authors:** Luca Wollenberg, Heiner Deubel, Martin Szinte

**Affiliations:** 1 Allgemeine und Experimentelle Psychologie, Ludwig-Maximilians-Universität München, Munich, Germany; 2 Graduate School of Systemic Neurosciences, Ludwig-Maximilians-Universität München, Planegg-Martinsried, Germany; 3 Department of Cognitive Psychology, Vrije Universiteit Amsterdam, Amsterdam, the Netherlands; McGill University, Canada

## Abstract

The premotor theory of attention postulates that spatial attention arises from the activation of saccade areas and that the deployment of attention is the consequence of motor programming. Yet attentional and oculomotor processes have been shown to be dissociable at the neuronal level in covert attention tasks. To investigate a potential dissociation at the behavioral level, we instructed human participants to move their eyes (saccade) towards 1 of 2 nearby, competing saccade targets. The spatial distribution of visual attention was determined using oriented visual stimuli presented either at the target locations, between them, or at several other equidistant locations. Results demonstrate that accurate saccades towards one of the targets were associated with presaccadic enhancement of visual sensitivity at the respective saccade endpoint compared to the nonsaccaded target location. In contrast, averaging saccades, landing between the 2 targets, were not associated with attentional facilitation at the saccade endpoint. Rather, attention before averaging saccades was equally deployed at the 2 target locations. Taken together, our results reveal that visual attention is not obligatorily coupled to the endpoint of a subsequent saccade. Rather, our results suggest that the oculomotor program depends on the state of attentional selection before saccade onset and that saccade averaging arises from unresolved attentional selection.

## Author summary

The premotor theory of attention postulates that spatial visual attention is a consequence of the brain activity that controls eye movement. Indeed, attention and eye movement share overlapping brain networks, and attention is deployed at the target of an eye movement (saccade) even before the eyes start to move. But is attention always deployed at the endpoint of saccades? Here, we measured visual attention before accurate saccades and before saccades that landed in between 2 targets (averaging saccades). While accurate saccades were associated with a selective enhancement of visual sensitivity at their endpoint, no such enhancement was found at the endpoint of averaging saccades. Rather, visual sensitivity was evenly distributed across the 2 saccade targets, suggesting that saccade averaging arises from unresolved attentional selection. Overall, our results reveal that attention is not always coupled to the endpoint of saccades, arguing against a simplistic view of the premotor theory of attention at the behavioral level. Instead, we propose that saccadic responses depend on the state of attentional selection at saccade onset.

## Introduction

To process information from our rich visual environment, we evolved with attentional mechanisms allowing us to discriminate which flow to account for and which to ignore [1,2]. For example, we can extract salient saccade targets from a cluttered visual scene to later examine their contents with precise foveal vision [3–6]. This link between attention and saccadic eye movements led researchers to propose that spatial visual attention is directly dependent on the oculomotor system [7,8], introducing what they called the “premotor theory of attention.”

This influential theory relies on 2 main hypotheses. The first hypothesis states that visual attention is operated by the oculomotor system itself. Indeed, overlapping neuronal activations have been observed in visual attention tasks involving the deployment of attention with (overt) or without (covert) eye movements in functional magnetic resonance imaging (fMRI) [9]. These activations include cortical and subcortical areas such as the Frontal Eye Field (FEF), the parietal cortex, and the Superior Colliculus (SC).

At the behavioral level, there is indeed evidence for a concurrent encoding of spatial attention and saccade programming [10]. For example, various studies demonstrated that visual attention, measured as a local improvement in visual sensitivity, is allocated to the saccade target before the eyes start to move [11,12]. Nevertheless, some other studies suggested that saccade preparation does not necessarily entail a shift of attention towards the saccade goal, casting some doubt in regard of the coupling between attention and oculomotor control [13–16].

The second hypothesis of the premotor theory of attention implies that the deployment of visual attention is always preceded by an activation of the oculomotor system. Under this hypothesis, covert attention involves the preparation of a saccade that is canceled before the eyes move. In line with this hypothesis, subthreshold microstimulation of the FEF or the SC, which did not systematically lead to a saccade, resulted in attentional benefits measured both behaviorally and electrophysiologically at the stimulated movement field position [17–20]. However, because microstimulation effects cannot be solely restricted to the motor cells within the stimulated areas, these results did not demonstrate that the deployment of visual attention is preceded by a premotor activation alone. Instead, it was shown that motor cells within FEF or SC stayed completely silent during a covert attention task [21–23], while visual and visuomotor cells displayed sustained attentional effects. In other words, attention is not always preceded by motor activity, at least not within these recorded oculomotor centers.

To shed light on this controversy and to test this second hypothesis at the behavioral level, one can imagine measuring visual sensitivity at the intended saccade goal and at the endpoint of the saccade. Under such conditions, measured sensitivity should correlate with the activity of both the visual and motor cells within oculomotor centers. Taking advantage of the fact that saccades tend to undershoot the target, Deubel and Schneider [12] found that attention was restricted to the intended saccade goal rather than to the saccade endpoint. However, using saccadic adaptation to decrease the saccadic gain, some authors found the exact opposite effect, with attention allocated to the adapted saccade endpoint rather than to the intended saccade goal [24,25]. Knowing that oculomotor centers have several overlapping large receptive fields within the range of these effects [26,27], it is hard to link these contradictory behavioral findings to the neurophysiology described above.

Here, we thus propose to use a paradigm leading to a larger spatial dissociation between the intended saccade goal and the saccade endpoint, such as the global effect [28–31]. Indeed, the global effect is associated with systematic and large saccade endpoint deviations towards the center of gravity of 2 saccade targets [28,32,33], or of a saccade target and a distractor [34,35], shown at 2 positions separated by up to 60° of rotation [34]. Although the global effect was originally described as reflecting a low-level averaging of neuronal activity (and therefore respective saccades are often called averaging saccades) within the oculomotor centers [28,36,37], different behavioral observations later suggested a dependency on higher-level attentional processes. First, it was shown that averaging saccades can be elicited by second- and third-order saccade targets [38,39], suggesting that the global effect cannot merely reflect low-level oculomotor processes. Next, it was shown that specifying the location [40,41], the identity [42,43], or the probability of a saccade target to appear at a certain location relative to a distractor [44] systematically reduced the occurrence of averaging saccades. Monkeys make averaging saccades when the FEF or the SC are simultaneously microstimulated at 2 sites [45–48] and when 2 targets are shown in close proximity [49,50]. At the neuronal level, it was first proposed that a single peak of motor cell activity associated with saccades ending in between 2 targets precedes an averaging saccade [51,52]. Later work suggested instead that averaging saccades follow 2 peaks of activity associated with saccades directed towards the 2 saccade targets [53,54]. Recently, Vokoun and colleagues [55] used voltage imaging of slices of rat SC to record population dynamics in response to dual-site electrical stimulation. They observed that the simultaneous stimulation of 2 nearby sites in the intermediate layers led to a merged peak centered in between them in the superficial layers. Moreover, they proposed that such merged activation feeds back into the visual system, leading to the perception of a target at the averaging saccade endpoint.

If this proposal of a feedback of merged activation from the superficial layers of the SC into the visual system was true, we would expect to find a presaccadic enhancement of attention at the endpoint of averaging saccades, a result that would be in line with the premotor theory of attention. Van der Stigchel and de Vries [56] directly tested this proposal, instructing participants to move their eyes towards a saccade target presented simultaneously with a distractor and measuring presaccadic attention at these positions as well as in between them. They observed both averaging saccades as well as saccades directed towards the target and the distractor, allowing them to compare the deployment of attention at the intended saccade goal and at the saccade endpoint. Unfortunately, they reported no main effect of the saccade landing direction as well as no interaction between the saccade landing direction and the position of their attention probes when analyzing visual discrimination performance as a function of the saccade endpoint. Therefore, contrary to many reports [11,12], the saccade landing position had no significant effect on the deployment of attention in their paradigm, preventing any conclusion about whether or not attention is deployed at the endpoint of averaging saccades.

Other studies suggested that attention is not necessarily allocated to the saccadic endpoint [11,44] or argued that saccades towards the center of gravity within extended target configurations are based on the computation of a central reference point via spatial pooling [57,58]. However, none of these studies measured visual attention at the averaging saccade endpoint to determine whether averaged oculomotor programs are associated with attentional averaging. Here, we measured visual attention at various locations in space, including the averaging saccade endpoint, in a free-choice saccade task that entailed the presentation of 2 nearby saccade targets. Our design therefore allowed us to investigate whether attention is allocated at the endpoint of averaging saccades. More specifically, given the spatial resolution of our design, we could distinguish the following 3 possible outcomes related to the deployment of visual attention before averaging saccades: (a) attention is deployed at the exact location of the saccade endpoint, (b) attention spreads across an extended area including the saccade endpoint, and (c) attention is deployed at 2 discrete saccade target areas flanking the saccade endpoint but not at the endpoint itself. We observed a presaccadic enhancement of visual sensitivity at the endpoint of accurate but not averaging saccades, ruling out an obligatory coupling of attention to the endpoint of a subsequently executed saccade (against [a]). Contrary to the idea of an extended spread of attention around the center of gravity, averaging saccades were associated with moderate enhancement of visual sensitivity at the 2 saccade targets (against [b]). Our results instead suggest that the oculomotor program depends on the state of attentional selection before saccade onset, with attention being deployed at the 2 discrete targets (favoring [c]) and saccade averaging resulting from uncompleted attentional selection.

## Results

**Fig 1:**
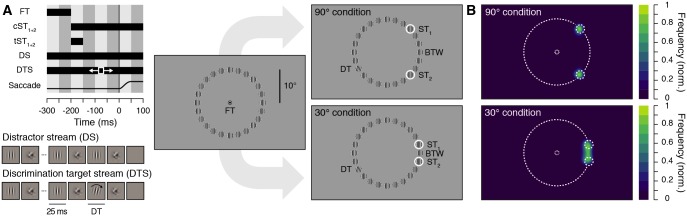
Experimental procedure and normalized saccade landing frequency maps. (A) Stimulus timing and display. Participants prepared a saccade from the fixation target to 1 of 2 potential saccade targets (ST_1_ and ST_2_), presented simultaneously at 2 randomly chosen stimulus streams with an intertarget angular distance of either 90° (top panels) or 30° (bottom panels). The saccade targets were either shown continuously (cST_1+2_) or transiently (tST_1+2_). Stimulus streams could either be distractor streams, composed of alternating vertical Gabors and masks (40 Hz), or discrimination target streams, which included the presentation of a brief discrimination target (25 ms)—a clockwise or counterclockwise tilted Gabor—shown between 75 and 175 ms after the saccade target onset. Participants saccaded towards 1 of the saccade targets and had to report the orientation of the discrimination target, appearing randomly at 1 of the 24 stimulus stream locations. Note that stimuli are sketched in order to increase their visibility. Actual stimuli match those shown in the stimulus streams depiction. (B) Normalized saccade landing frequency maps averaged across participants (*n* = 10) for the 90° (top) and 30° (bottom) conditions (collapsed across the transient and continuous saccade target presentation). BTW, position in between the saccade targets; cST_1+2_, saccade targets continuously shown; DS, distractor stream; DT, discrimination target; DTS, discrimination target stream; FT, fixation target; ST_1_, saccade target 1; ST_2_, saccade target 2; tST_1+2_, saccade targets transiently shown.

Our goal was to determine whether the presaccadic deployment of attention is obligatorily coupled to the saccade endpoint. To do so, we probed visual attention at various locations while participants prepared a saccade towards 1 of 2 potential saccade targets, presented either transiently or continuously and separated by an intertarget angular distance of either 90° or 30° (Fig 1A). Just before the saccade, a discrimination target was shown randomly across trials at 1 of the 2 potential saccade targets (ST_1_ and ST_2_), at the position in between the saccade targets (BTW), or at 1 of 21 equidistant control positions (CTRL).

**Fig 2:**
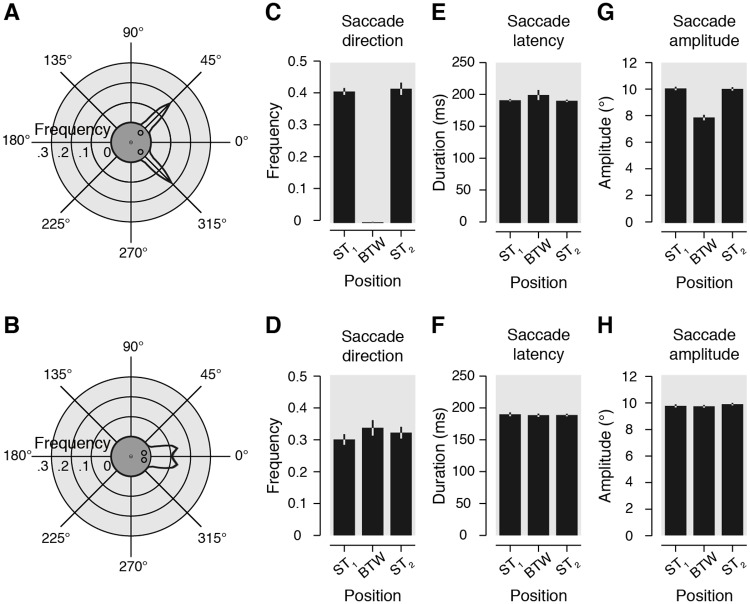
Saccade metrics. (A, B) Circular plots show the averaged frequency distribution of the saccade landing direction binned in evenly distributed angular sectors of 5°, in the 90° (panel A) and 30° (panel B) conditions. Stimulus configuration is rotated as to align the 2 saccade targets symmetrically around the geometrical angle zero (see central insets). (C, D) Bar graphs illustrate averaged frequency of trials as a function of the saccade landing direction binned in 24 evenly distributed angular sectors of 15°. Data are shown for the 3 positions of interest (ST_1_, BTW, and ST_2_) in the 90° (panel C) and 30° (panel D) conditions. (E–H) Averaged saccade latency (E, F) and amplitude (G, H) observed for the same 3 positions of interest in the 90° (panel E and G) and 30° conditions (panel F and H). All data are shown irrespective of the duration (continuously or transiently) of the saccade targets. Light gray areas and error bars represent SEM. Polar plot black lines and corresponding light gray areas show linear interpolation between data points. BTW, position in between the saccade targets; ST_1_, saccade target 1; ST_2_, saccade target 2.

Fig 1B shows the normalized frequency of saccade landing endpoints observed across participants within the 90° and 30° condition, irrespective of the duration of the saccade targets (i.e., transient and continuous combined). While saccades were equally distributed over the 2 saccade targets in the 90° condition (Fig 1B, top), a substantial proportion of saccades ended in between them in the 30° condition (Fig 1B, bottom). To further analyze our data, we looked at the distribution of saccade landing directions either binned in evenly distributed angular sectors of 5° (Fig 2A and 2B) or 15° (centered on the 24 stimuli streams, Fig 2C and 2D). In the 90° condition (Fig 2C), 41.0% ± 1.0% of the saccades ended within the sector including ST_1_ (most counterclockwise saccade target) and 41.8% ± 1.9% within the sector including ST_2_ (most clockwise saccade target). Note that an average of 4.0% ± 0.9% of saccades ended within the sectors adjacent to the saccade targets. In the 30° condition (Fig 2D), 33.6% ± 2.4% of the saccades ended within the sector in between the 2 saccade targets (BTW), while 29.95 ± 1.6% of the saccades ended within the sector of ST_1_ and 32.0% ± 1.8% within the sector of ST_2_. Therefore, when participants had to select between 2 equidistant saccade targets separated by an angular distance of 30°, they executed an averaging saccade (ending in the BTW sector) in about one-third of the trials. For further inspection, saccade endpoint distributions as a function of saccade latency are provided for each participant in S1 Fig. In order to determine potential differences between the 2 intertarget angular distance conditions (90° and 30°), we first looked at saccade latencies and amplitudes. We found slightly longer saccade latencies (90°: 192.2 ± 1.7 ms versus 30°: 188.2 ± 2.2 ms; *p* = 0.0012) and larger amplitudes (90°: 10.0 ± 0.1° versus 30°: 9.7 ± 0.1°; *p* = 0.0002) in the 90° as compared to the 30° condition. Saccade latency did not differ as a function of the saccade landing position (ST_1_, ST_2_, or BTW) both in the 90° and 30° condition (all *p* > 0.05, Fig 2E and 2F). In the 90° condition, amplitudes of saccades towards ST_1_ (10.1 ± 0.1°) and ST_2_ (10.0 ± 0.1°) did not differ significantly from each other (ST_1_ versus ST_2_: *p* = 0.7902), whereas amplitudes of saccades towards BTW (7.9 ± 0.2°) were significantly smaller than those of saccades towards ST_1_ and ST_2_ (both *p* < 0.0001) (see Fig 2G). In the 30° condition, amplitudes of saccades towards ST_1_ (9.7 ± 0.1°) and ST_2_ (9.8 ± 0.1°), as well as towards ST_1_ and BTW (9.7 ± 0.1°), did not differ significantly from each other (ST_1_ versus ST_2_: *p* = 0.2216; ST_1_ versus BTW: *p* = 0.5998), whereas amplitudes of saccades towards ST_2_ were significantly larger than those of saccades towards BTW (ST_2_ versus BTW: *p* = 0.0118) (see Fig 2H). Note that the proportion of averaging saccades did not vary as a function of saccade latency. Comparing trials of the 30° condition separated in 2 equal groups of early (167.1 ± 1.8 ms) and late (209.3 ± 3.2 ms) saccade latencies, we found a comparable proportion of averaging saccades (early BTW: 35.1 ± 3.0% versus late BTW: 32.1 ± 2.2%; *p* = 0.1632). This effect is most likely the consequence of the instruction given to the participants to saccade as fast as possible, such that early and late averaging saccade latencies differed by less than 40 ms (early BTW: 168.2 ± 2.0 ms versus late BTW: 207.4 ± 3.1 ms; *p* < 0.0001). However, we found that the mean absolute saccade endpoint deviation relative to the BTW location slightly increased as a function of saccade latency (see A-B in S2 Fig and A-B in S2 Fig for individual participant data for both the 90° and 30° conditions). Thus, saccade averaging was more pronounced for short-latency saccades. Overall, for each intertarget angular distance, we observed either no differences or only some nonsystematic differences of a few milliseconds and a few minutes of arc. Although saccade latencies and amplitudes did not differ much between these conditions, the saccade landing-direction distributions reflect 2 distinct oculomotor modes as a function of the intertarget angular distance. Saccades were mostly accurate in the 90° condition, whereas we observed both accurate and averaging saccades in the 30° condition.

**Fig 3:**
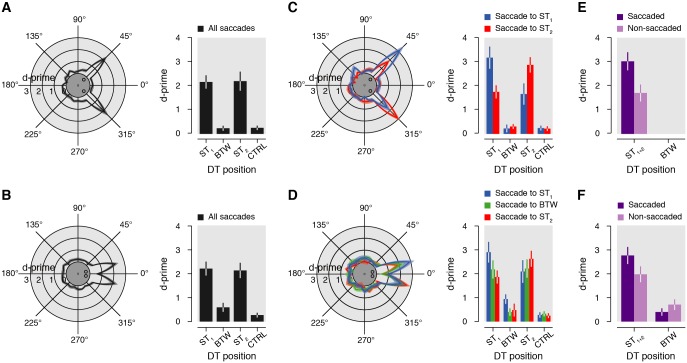
Visual sensitivity. (A, B) Circular plots show averaged visual sensitivity (d’) as a function of the DT position in the 90° (panel A) and 30° (panel B) conditions, irrespective of the duration of the saccade targets and across all saccade directions observed. Bar graphs illustrate visual sensitivity for 4 positions of interest (ST_1_, BTW, ST_2_, CTRL). (C, D) Visual sensitivity as a function of the DT position relative to the saccade landing direction in the 90° (panel C) and 30° (panel D) conditions, irrespective of the duration of the saccade targets (blue: saccade to ST_1_; green: saccade to BTW; red: saccade to ST_2_). For each saccade direction, we took the average sensitivity for each discrimination target location. For example, the blue line plots visual sensitivity when saccades were made towards ST_1_ and the discrimination target was either at ST_1_ (+15° on the polar plot), BTW (15° counterclockwise to ST_1_; 0° on the polar plot), or ST_2_ (30° counterclockwise to ST_1_; +345° on the polar plot), and so on. (E, F) Bar graphs illustrate sensitivity observed for DT shown at the saccaded (purple: e.g., DT at ST_1_ and saccade to ST_1_) and the nonsaccaded (light purple: e.g., DT at ST_1_ and saccade to ST_2_ or BTW) positions in the 90° (panel E) and the 30° (panel F) conditions. Conventions are as in Fig 2. BTW, position in between the saccade targets; CTRL, control position; DT, discrimination target; ST_1_, saccade target 1; ST_2_, saccade target 2.

Our paradigm allowed us to measure both the oculomotor behavior and the presaccadic allocation of attention through the presentation of a discrimination target at 1 of 24 possible positions. We first verified that the presentation of the discrimination target itself did not systematically influence oculomotor behavior. We did not find any differences with respect to saccade latency and amplitude when comparing trials with and without the presentation of a discrimination target (3.5% of trials were without discrimination target, both *p* > 0.05). This result validates that the distractor streams and, in particular, the presentation of a discrimination target did not bias the deployment of attention. Fig 3A and 3B shows visual sensitivity as a function of the discrimination target position rotated as to align the 2 saccade targets around the geometrical angle zero in both the 90° (Fig 3A) and 30° (Fig 3B) condition. Irrespective of the duration of the saccade targets, we found higher sensitivity for discrimination targets shown at the saccade targets than at the control positions (corresponding to the average across all positions except for ST_1_, ST_2_, and BTW) in both the 90° (ST_1_: d’ = 2.2 ± 0.3 versus CTRL: d’ = 0.3 ± 0.1, *p* < 0.0001; ST_2_: d’ = 2.2 ± 0.4 versus CTRL, *p* < 0.0001; ST_1_ versus ST_2_, *p* = 0.8964; Fig 3A) and the 30° (ST_1_: d’ = 2.2 ± 0.3 versus CTRL: d’ = 0.3 ± 0.1, *p* < 0.0001; ST_2_: d’ = 2.1 ± 0.3 versus CTRL, *p* < 0.0001; ST_1_ versus ST_2_, *p* = 0.6026; Fig 3B) condition. These effects contrast with the low sensitivity observed for discrimination targets shown in between the saccade targets (BTW) in the 90° (BTW: d’ = 0.2 ± 0.1 versus ST_1_, *p* < 0.0001; BTW versus ST_2_, *p* < 0.0001) and especially in the 30° (BTW: d’ = 0.6 ± 0.2 versus ST_1_, *p* < 0.0001; BTW versus ST_2_, *p* < 0.0001) condition.

Thus, despite the fact that saccades landed in between the saccade targets in a third of the trials in the 30° condition, the overall sensitivity at this position stayed rather low. One should, however, note that sensitivity was still increased at this position compared to the control positions in the 30° condition (30°: BTW versus CTRL, *p* = 0.0010), whereas this was not the case in the 90° condition (90°: BTW versus CTRL, *p* = 0.7732). On the other hand, such slight facilitation observed in between the saccade targets in the 30° condition relative to the control positions was only observed for trials in which the targets were shown transiently (BTW: d’ = 0.8 ± 0.2 versus CTRL: d’ = 0.3 ± 0.1, *p* < 0.0001) but not continuously (BTW: d’ = 0.5 ± 0.2 versus CTRL: d’ = 0.3 ± 0.0, *p* = 0.10880). It is important to note that the discrimination target temporally overlapped with the saccade targets in the continuous but never in the transient condition. The observed difference between the 2 conditions therefore suggests that the appearance of a discrimination target at BTW was masked by the continuous presentation of the saccade targets. Altogether, the results above demonstrate that presaccadic attention was mainly allocated towards the saccade targets, and to a much smaller extent towards the position in between. This last result, however, cannot be attributed to a large spread of attention extending to more than 1 of the tested directions because we did not observe a consistent benefit at the 2 other positions adjacent to the saccade targets in the 30° condition (ST_1_ + 15°: d’ = 0.4 ± 0.1 versus CTRL: d’ = 0.3 ± 0.1, *p* = 0.0914; ST_2_ − 15°: d’ = 0.4 ± 0.1 versus CTRL, *p* = 0.0336; here, CTRL excludes ST_1_ + 15° and ST_2_ − 15°, respectively, in addition to ST_1_, ST_2_, and BTW) nor at the 4 adjacent positions of the saccade targets in the 90° condition (ST_1_ ± 15°: d’ = 0.3 ± 0.1 versus CTRL: d’ = 0.2 ± 0.1, *p* = 0.5742; ST_2_ ± 15°: d’ = 0.3 ± 0.1 versus CTRL, *p* = 0.3200; here, CTRL excludes ST_1_ ± 15° and ST_2_ ± 15°, respectively, in addition to ST_1_, ST_2_, and BTW).

At that stage, one cannot exclude the possibility that attention is always drawn towards the saccade endpoint before both accurate and averaging saccades because we found higher sensitivity for both the saccade targets—and, in the 30° condition, also for the position in between them—compared to the control locations. Although we found higher sensitivity at the saccade targets than in between them, this may just reflect the combined effect of the saccade preparation and the presence of visual cues (the saccade targets themselves). To estimate the effect of saccade preparation, we thus needed to specify our results depending on where the saccade ended within each trial. To do so, we redefined the position of the discrimination targets relative to the saccade direction. Fig 3C and 3D shows visual sensitivity as a function of the discrimination target position relative to the saccade direction. We found higher sensitivity for discrimination targets shown at the saccade targets when compared to the position in between them or to the control positions in both the 90° and 30° conditions, for trials in which accurate saccades were made towards ST_1_ (all *p* < 0.0001) or ST_2_ (all *p* < 0.0001). The same effects were found for averaging saccades in the 30° condition (all *p* = 0.00010). In addition to the facilitation effect of the saccade target presentation, we found that, irrespective of the intertarget distance (90° or 30°), sensitivity at ST_1_ was improved when an accurate eye movement was made towards ST_1_ (90°: ST_1_: d’ = 3.2 ± 0.5 versus ST_2_: d’ = 1.7 ± 0.4, *p* < 0.0001 [see blue lines and bars in Fig 3C and 3D]; note that in the 30° condition, sensitivity at ST_1_: d’ = 2.9 ± 0.4 was only marginally superior to those observed at ST_2_: d’ = 2.1 ± 0.5, *p* = 0.0740). The same selective improvement was observed at ST_2_ before the execution of accurate saccades towards it (90°: ST_2_ versus ST_1_, *p* < 0.0001; 30°: ST_2_ versus ST_1_, *p* = 0.0002 [see red lines and bars in Fig 3C and 3D]). In particular, preparing an accurate eye movement towards 1 of the 2 saccade targets improved sensitivity when comparing trials in which the discrimination target was shown at the saccaded location (e.g., DT at ST_1_ and saccade made towards ST_1_) to trials in which the discrimination target was shown at the same position when it was not the saccaded position (e.g., DT at ST_1_ and saccade landing at ST_2_ or BTW) in both the 90° (Fig 3E; ST_1+2_ saccaded: d’ = 3.0 ± 0.4 versus ST_1+2_ nonsaccaded: d’ = 1.7 ± 0.4, *p* < 0.0001) and the 30° (Fig 3F; ST_1+2_ saccaded: d’ = 2.7 ± 0.4 versus ST_1+2_ nonsaccaded: d’ = 2.0 ± 0.3, *p* = 0.0080) condition. Crucially for averaging saccade trials, for which the intended saccade goal (ST_1_ or ST_2_) and the saccade endpoint (BTW) were dissociated (see green lines and bars in Fig 3D), we found a rather low sensitivity for discrimination targets shown in between the saccade targets (BTW: d’ = 0.4 ± 0.2), highly reduced when compared to discrimination targets shown at the saccade targets (ST_1_: d’ = 2.2 ± 0.4 and ST_2_: d’ = 2.2 ± 0.4, both *p* < 0.0001). Furthermore, and contrary to above (Fig 3B), it was not different from the sensitivity gathered across the control locations (CTRL: d’ = 0.3 ± 0.1, *p* = 0.4026), both when the saccade targets were shown transiently or continuously (both *p* > 0.05). Thus, contrary to accurate saccades, the execution of averaging saccades did not lead to any improvement at the saccade endpoint. Moreover, a visual inspection of sensitivity as a function of the saccade latency shows a relative independence of these measures, suggesting that, irrespective of the saccade latency, attention was not deployed at the averaging saccade endpoint (see C-D in S2 Fig and A-B in S4 Fig for individual participant data in the 90° and 30° conditions). Visual sensitivity was significantly reduced at the intermediate location (BTW) before averaging saccades compared to saccades that landed at 1 of the saccade targets (Fig 3F; BTW saccaded: d’ = 0.4 ± 0.2 versus BTW nonsaccaded: d’ = 0.7 ± 0.2, *p* < 0.0001). This sensitivity reduction can, however, be mainly attributed to a masking effect of the continuous presentation of the saccade targets (BTW saccaded: d’ = 0.3 ± 0.3 versus BTW nonsaccaded: d’ = 0.7 ± 0.2, *p* = 0.0088) because it was not found for saccade targets presented transiently (BTW saccaded: d’ = 0.7 ± 0.2 versus BTW nonsaccaded: d’ = 0.7 ± 0.3, *p* = 0.9664).

These findings demonstrate, contrary to what is predicted by the premotor theory of attention, that the preparation of averaging saccades does not lead to a deployment of attention at the corresponding saccade endpoint. Instead, we found that averaging saccades were associated with an equal distribution of attention towards the 2 saccade targets (ST_1_: d’ = 2.2 ± 0.4 versus ST_2_: d’ = 2.2 ± 0.4, *p* = 0.8402). One interpretation of these effects could be that averaging saccades result from an unsuccessful or at least uncompleted presaccadic attentional selection among the 2 saccade targets, with resources equally distributed between them. On the other hand, it is possible that, despite landing in between the targets, presaccadic attentional selection was successful before averaging saccades but directed half of the time towards the most clockwise saccade target and half of the time towards the most counterclockwise saccade target. If this were the case, across trials, one would also expect to find an equal and moderate enhancement of sensitivity for discrimination targets shown at the saccade targets.

To disentangle these 2 interpretations, we analyzed trials in which a corrective saccade followed the execution of an averaging saccade. We reasoned that if averaging saccades resulted from a successful trial-by-trial presaccadic attentional selection of 1 of the 2 saccade targets, they should be followed by corrective saccades directed equally often towards both targets. Moreover, they should be associated with an attentional benefit at the goal of the corrective saccades. Contrary to these predictions, we observed corrective saccades in only 48.1% ± 5.8% of the averaging saccade trials. Corrective saccades were not all clearly directed towards the saccade targets (see A-B in S5 Fig), ending either in the angular sector of the most counterclockwise saccade target (ST_1_: 48.3% ± 3.1% of all the corrective saccades following an averaging saccade), the most clockwise saccade target (ST_2_: 38.3% ± 2.5%), or in between them (BTW: 11.9% ± 2.8%). They were, moreover, not equally often directed towards each of the saccade targets (ST_1_ versus ST_2_, *p* = 0.0288), probably reflecting a bias of our participants. As shown in C in S5 Fig, we did not find any significant benefit at the endpoint of the corrective saccades following an averaging saccade, when comparing trials in which discrimination targets were shown at the endpoint of the corrective saccade (ST_1+2_ correctively saccaded: d’ = 2.8 ± 0.5) to trials in which a discrimination target was shown at the same position when it was not the endpoint of the corrective saccade (ST_1+2_ correctively nonsaccaded: d’ = 2.5 ± 0.8, *p* = 0.68300). Moreover, no significant benefit could be found when the corrective saccades following an averaging saccade ended still in between the saccade targets (BTW correctively saccaded: d’ = 0.7 ± 1.1 versus BTW correctively nonsaccaded: d’ = −0.1 ± 0.6, *p* = 0.4698). Taken together, these results suggest that averaging saccades result from an unsuccessful or uncompleted presaccadic attentional selection among the 2 saccade targets.

Finally, we wanted to exclude the possibility that the poor discrimination performance at the endpoint of averaging saccades was a result of the rather coarse saccade direction binning used in our analysis (±7.5° of rotation around ST_1_, BTW, ST_2_, and the distractor locations). We chose this binning procedure to end up with 24 equal saccade direction bins centered on the locations at which we measured visual sensitivity. Nevertheless, one might argue that we thereby classified a substantial proportion of saccades as averaging saccades (landing within the BTW bin) despite the possibility that they were actually biased towards 1 of the saccade targets and landed in the outer areas of the bin. To validate our analysis, we analyzed visual sensitivity as a function of the saccade direction using smaller bins (±2.5°). As evident in S6 Fig, in which we contrast the data for these 2 binning procedures, the smaller binning did not systematically alter our results. Crucially, we still found low visual sensitivity at BTW even for the proportion of saccades landing precisely at the most central bin (i.e., within ±2.5° around the center of BTW).

## Discussion

We observed a clear oculomotor dissociation between trials in which 2 equidistant saccade targets were shown at 2 different angular distances from each other. While only accurate saccades were found for an intertarget angular distance of 90°, we observed both accurate and averaging saccades when the same targets were separated by 30°. Combined with a measure of presaccadic visual sensitivity, this dissociation allowed us to determine the influence of saccade preparation on the deployment of attention when the intended saccade goal and the saccade endpoint were spatially associated (accurate saccades) or clearly dissociated from each other (averaging saccades). Accurate saccades were associated with a strong and systematic presaccadic enhancement of visual sensitivity at the saccade endpoint when compared to the nonsaccaded locations for intertarget angular distances of both 90° and 30°. In contrast, we did not observe a presaccadic enhancement of visual sensitivity at the endpoint of averaging saccades. Rather, averaging saccades were associated with an equal deployment of attention at the 2 saccade target locations. Our corrective saccade analysis indicated that this result cannot be explained by a trial-by-trial presaccadic attentional selection of 1 of the 2 saccade targets. Overall, these effects rule out the proposal that the deployment of attention is strictly derived from the upcoming oculomotor program. Rather, they reflect a spatial dissociation between the deployment of visual attention and the averaging saccade endpoint. More specifically, these results rule out an account in which attention is precisely allocated to the saccade endpoint (alternative [a] in Introduction) or spreads over an extended region including the saccade endpoint before averaging saccades (alternative [b] in Introduction). Our data instead favor an account in which attention is equally allocated at 2 discrete saccade target locations before averaging saccades (alternative [c] in Introduction). Contrary to the idea that the activation of the oculomotor system precedes spatial attention, we propose that the oculomotor program depends on the state of attentional selection before the saccade, with averaging saccades arising from an uncompleted attentional selection process.

Findlay [28] referred to the "global effect" as the phenomenon of directing the eyes towards the center of gravity of 2 presented targets [29]. To his view, this phenomenon reflects a coarse or global processing of a visual scene before rapidly generated eye movements. His account thus predicts that in our experiment, visual sensitivity should be coarsely distributed over the 2 saccade targets as well as over their adjacent locations before the execution of averaging saccades. Our precise measure of presaccadic visual sensitivity allowed us to determine the spatial specificity of attentional deployment during saccade preparation. Contrary to the notion of a global processing (including the locations at the saccade targets and in between) before averaging saccades, we observed a precise allocation of attention limited only to the saccade targets (limited to at least approximately 2.6°, the distance between 2 of our adjacent stimuli). Therefore, before an averaging saccade, the visual system indeed seems to have precise access to the saccade target configuration, reflecting an enhancement of local rather than global visual information processing [59]. Such a discontinuous deployment of attention was also found in various tasks entailing the presentation of multiple targets [60–62]. Our results can also rule out other models of averaging saccades based solely on low-level oculomotor processing [36,37,63]. We report here that when an accurate saccade is prepared towards 1 of 2 identical saccade targets, the subsequent movement correlates with an attentional benefit at the saccade endpoint, whereas averaging saccades resulted in the absence of a selective attentional benefit at 1 of the 2 targets as well as in between them (i.e., at the saccade endpoint). In this regard, our results match with previous studies showing a reduction in the occurrence of averaging saccades when attentional selection of the saccade goal is made easier by specifying its location or its identity [40–44]. Similarly, a model relying on attentional selection could also explain why averaging saccades are less often observed in delayed saccade tasks [40,64], as they also give more time for the attentional selection to complete [43]. Early studies have often reported that averaging saccades are associated with faster saccade latencies as compared to accurate saccades [28,34]. Yet, recently, Weaver, Zoest, and Hickey [65] proposed that the spatial and temporal components of saccade programming are relatively independent from each other. They argued that attentional mechanisms can affect oculomotor behavior only when acting upon it before the onset of the movement. It might well be that our instructions to saccade as fast and as accurately as possible reduced the saccade latency range and thereby reduced potential differences between the latencies of accurate and averaging saccades. Furthermore, given that participants were engaged in a dual task, the attentional task might have slowed down saccade execution, leading to averaging saccades even at longer latencies. We propose that the type of saccade executed on a given trial was determined by the speed at which attentional selection was processed. Accordingly, accurate saccades were presumably executed whenever attentional selection of a target was readily resolved before saccade onset.

Another account of the global effect is that averaging saccades reflect a time-saving strategy [40], in which an averaging saccade followed by a correction movement allows for faster oculomotor action than a deliberately delayed accurate saccade. Given that participants saccaded accurately towards one of the targets with a similar latency as found for averaging saccades in two-thirds of the trials in our paradigm, our results speak against such a strategy. Although we observed some corrective saccades that ended nearby the saccade targets and therefore increased the accuracy of initial averaging saccades, they came with a cost of about 200 ms, rendering such strategy inefficient. Moreover, if participants would have strategically planned 2 successive saccades (an averaging saccade followed by a corrective saccade), we would expect to find attentional benefits at both saccade endpoints as reported in sequential saccade tasks [62,66]. Contrary to this prediction, we found neither an attentional enhancement at the endpoint of averaging saccades nor at the endpoint of corrective saccades compared to the positions not reached by corrective saccades.

Therefore, our results argue against earlier accounts of the global effect and propose that averaging saccades reflect a compromise between the dynamics of attentional selection and the instructions to move the eyes as fast as possible. Our proposal is based on the results of a combined measure of visual attention and averaging saccades. Similar to a previous report [56], we found an overall enhancement of visual sensitivity at the 2 saccade targets, when the data were not split depending on the saccade direction. In order to conclude on the deployment of attention before averaging saccades, however, one needs to specify visual sensitivity depending on the saccade direction. Crucially, and contrary to Van der Stigchel and de Vries [56], we indeed found an influence of the saccade direction (i.e., endpoint) on the allocation of attention when taking into account saccade direction. Within a paradigm producing both accurate and averaging saccades, we observed a presaccadic shift of attention [11,12], reflected by selectively enhanced sensitivity at the endpoint of accurate saccades. The replication of this presaccadic attention effect comes as a prerequisite to drawing conclusions on the effect of averaging saccades, for which, instead, we found no attentional benefit at the saccade endpoint. Van der Stigchel and de Vries [56] concluded that there is no attentional shift towards the endpoint of averaging saccades. However, they also reported no main effect of the saccade landing direction as well as no interaction between the saccade landing direction and the position of their attention probes when they analyzed their data as a function of the saccade endpoint. Their results are therefore inconclusive, or even speak in favor of an attentional global effect. Moreover, when we combined all trials irrespective of the saccade direction, we found a slight increase of sensitivity at the position in between the 2 potential saccade targets when they were presented transiently but not when they were presented continuously. Because Van der Stigchel and de Vries [56] used a continuous presentation of a saccade target and a distractor, their results most likely reflect a masking effect of their stimuli on the discrimination target rather than an absence of attentional modulation. Here, we clearly dissociated attention allocated to the intended saccade goal from attention allocated to the endpoint of the saccade and found no benefit at the averaging saccade endpoint. This result is theoretically consistent with the idea that attention is not restricted to the endpoint of a saccade [11,44] and provides behavioral evidence against the main hypothesis of the premotor theory of attention, which postulates that the deployment of visual attention is derived from oculomotor programming [7,8].

**Fig 4:**
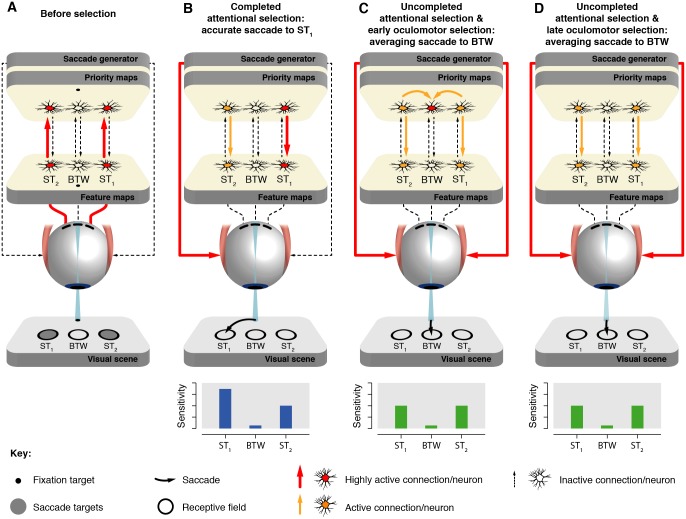
Attentional selection determines saccade endpoint. Two nearby saccade targets (gray dots) are flashed in the periphery from the fixation target and projected onto the retina, triggering a cascade of bottom-up (upward arrows) and top-down (downward arrows) processes throughout the visual processing hierarchy. Colors of the neurons and arrows indicate the level of activation. Each retinal projection connects to a specific neuron (in fact, a population of neurons) in retinotopic feature maps (V1–V4, MT). Feature map neurons, in turn, are linked to priority maps (FEF, LIP, SC). Priority map activity is later integrated by the saccade generator (brainstem) driving the extra-oculomotor muscles. Note that the priority maps and the saccade generator are distinct components within the processing hierarchy. The data panels show the predicted sensitivity at the saccade targets (ST_1_ and ST_2_) and in between them (BTW), and curved black arrows show the predicted saccade path. (A) Before attentional selection, ST_1_ and ST_2_ neuronal columns are highly activated by bottom-up connections driven by the saccade target onset. (B) Following a decay in the activity of both ST_1_ and ST_2_ neuronal columns, a completed attentional selection leads to a high activation of either ST_1_ or ST_2_ neurons in the priority maps (in this example, ST_1_ is selected), propagating via top-down connections to the feature maps. This leads to a presaccadic enhancement of sensitivity at the selected target and subsequently to an accurate saccade towards it (in this example, towards ST_1_). (C, D) Uncompleted attentional selection leads to an equal and moderate presaccadic sensitivity enhancement at the saccade targets, but not in between. Subsequently, the bimodal collicular activity distribution merges into a unimodal distribution around the intermediate collicular site (panel C) or remains bimodal and is later integrated by the saccade generator (panel D). In either case, an averaging saccade is executed towards the intermediate location (BTW). BTW, position in between the saccade targets; DT, discrimination target; FEF, Frontal Eye Field; LIP, Lateral Intraparietal Cortex; MT, Middle Temporal Visual Area; SC, Superior Colliculus; ST_1_, saccade target 1; ST_2_, saccade target 2; V, Visual Area.

We illustrate our results in a theoretical framework (Fig 4), inspired by both behavioral and neurophysiological findings, linking visual attention and oculomotor programming [67]. This theoretical framework neither provides a strict model nor a computational framework. It aims at putting our results in the context of the current view on saccade programming and yielding new testable predictions. We propose that our attentional effects rely on a top-down modulation [5,19] of feature-selective areas of the visual cortex by the priority maps [68]. Initially, the onsets of the saccade targets strongly activate neurons with corresponding receptive fields within columns of the feature and priority maps (Fig 4A). Their activity will then decay until the saccade target-selection process begins. We propose that, before an accurate saccade, one of the saccade targets is selected, such that oculomotor cells centered on the saccaded location become more active in comparison to those encoding the nonsaccaded target location (Fig 4B). Because our 2 targets were physically identical, saccade target selection probably occurs within the priority maps and propagates via a top-down mechanism to the corresponding feature map columns [5,69–71]. Oculomotor cells within the priority maps are connected to the areas of the brainstem circuitry controlling the horizontal (e.g., pons and medulla) and vertical (e.g., rostral midbrain) components of an eye movement [72,73]. Given that only 1 saccade can be executed at a time, a winner-takes-all integration of the motor output [47,74,75] from the priority maps is typically assumed such that the most active population will determine the subsequent saccade vector. The exact nature of this integration is, however, beyond the scope of this study. Thus, in our framework, an accurate saccade towards the selected saccade target (i.e., the saccade target that is represented as the most active population at the level of the priority maps) is triggered by the saccade generator, and the activity state within the feature maps leads to higher sensitivity at the saccade endpoint before the eyes start to move (Fig 4B).

Following the same rationale, we propose that averaging saccades arise from an unresolved saccade target-selection process. Given the behavioral nature of our data, we can only speculate about the neural correlates of averaging saccades at the level of the priority maps in this experiment. We will, however, discuss our results in the light of 2 alternative accounts concerning the representation of averaging saccades at the level of the SC. While Edelman and Keller [54] found evidence for a bimodal distribution of collicular activity before averaging saccades at express latencies, an earlier study by Glimcher and Sparks [52] argued for an intermediate unimodal distribution in case of regular-latency averaging saccades.

Because averaging saccades were executed at regular latencies in this experiment, they might indeed have been associated with a unimodal distribution of activity at an intermediate collicular site (early oculomotor selection—Fig 4C) at saccade onset. According to this view, averaging saccades were initially reflected by 2 equally enhanced collicular populations coding for the 2 saccade targets. This bimodal distribution of activity propagates to the feature maps, leading to an equal enhancement of visual sensitivity at the 2 saccade targets. However, the initial bimodal collicular activity distribution then progresses into a unimodal distribution centered at an intermediate collicular site to subsequently allow for the execution of a single saccade. Such a scenario is in line with evidence from a recent study performing dual-site electrical stimulation in the intermediate layers of the SC [55]. If the absence of attentional deployment at the averaging saccade endpoint observed here was indeed associated with a single active population located at an intermediate site of the SC, our results would clearly refute the premotor theory of attention.

Alternatively, averaging saccades may result from a bimodal collicular activity distribution at saccade onset (late oculomotor selection—Fig 4D). In this case, the collicular sites of enhanced activity would match with the observed attentional benefits at the 2 saccade targets, and oculomotor averaging across the active collicular populations would be achieved by integration downstream of the SC. This conception could be considered compatible with a weak version of the premotor theory of attention because one could argue that the output from the SC—which is likely the last node for visuomotor transformation—is simultaneously recruited to guide attention and eye movements. However, while the final oculomotor program was averaged, attention clearly was not in this experiment. Thus, attentional and oculomotor programming are necessarily dissociable at some processing level. One possible option to account for the observed dissociation at the behavioral level is to assume that the brainstem circuitry and the attentional system deploy different algorithms to read out the collicular code.

Disentangling the 2 options discussed above (early versus late oculomotor selection) would constitute an important step in the understanding of the link between attention and action and would require simultaneous behavioral and neural recordings. In regard to the neural recording, one should, however, carefully distinguish between the different classes of neurons (fixation, visual, motor, and visuomotor), which appear to reside along a continuum with variable response properties depending on the experimental conditions [76].

According to our view, attentional selection is not completed at the onset of averaging saccades, as reflected by the equal and moderate attentional benefits at the saccade targets. This proposal is supported by electrophysiological recordings showing that averaging saccades are associated with 2 distinct peaks within the intermediate layers of the SC [53,54]. A similar, general conception of oculomotor programming was expressed by He and Kowler [44], who proposed a 2-stage process in which a single mechanism resolves attentional selection before the oculomotor program is computed at a later stage based on attentional weighting. Our results, moreover, go against a recent proposal that a merged activation within the superficial layers of the SC would feed back into the visual system [55] because this should have led to some attentional enhancement in between the saccade targets before an averaging saccade.

Our framework leads to some predictions in regard to the global effect. First, it predicts that any experimental manipulation modifying the difficulty of saccade target selection will directly impact the occurrence of averaging saccades. For example, specifying the location, the identity, or the probability of a saccade target appearing at a certain location will decrease the task difficulty, thereby increasing the speed of the attentional selection process and reducing the occurrence of averaging saccades [40–44,77]. Also, it predicts that, at a given latency, an easy saccade task should lead to fewer averaging saccades as compared to a more difficult one. Using a simple 2-saccade target task, it was shown that monkeys make averaging saccades only for express but not for normal saccade latencies [50], whereas they execute averaging saccades even for normal saccade latencies in a task rendered harder by a visual search display [49]. Similarly, Viswanathan and colleagues [78] showed that—at a saccade latency for which no consistent global effect was found with a distractor shown nearby a prosaccade target—a clear global effect was evident with the same distractor shown nearby an antisaccade target. These results are in line with our first prediction, as antisaccades are associated with a slower attentional selection [79]. Second, our framework predicts that one should not find any incremental presaccadic attentional benefit at one of the competing saccade targets before an averaging saccade, irrespective of the observed saccade latency. Future studies could directly test this prediction by measuring neuronal activity associated with the saccade targets before an averaging saccade. Third, we proposed 2 alternative explanations that could account for the observed behavioral dissociation between attention and the saccade endpoint before averaging saccades at the neuronal level. Both accounts question the validity of the premotor theory of attention in a saccade task rather than in a covert attention task [21–23].

Combining a measure of presaccadic visual sensitivity with a free-choice saccade task, we spatially dissociated attention allocated to the intended saccade goal from attention allocated to the saccade endpoint. We report here that attention is not obligatorily coupled to the endpoint of the oculomotor program, providing evidence against the strict view that oculomotor processes precede attention. Instead, we propose that saccadic responses depend on the state of attentional selection at saccade onset.

## Materials and methods

### Ethics statement

This experiment was approved by the Ethics Committee of the Faculty for Psychology and Pedagogics of the Ludwig-Maximilians-Universität München (approval number 13_b_2015) and conducted in accordance with the Declaration of Helsinki. All participants gave written informed consent.

### Participants

Thirteen participants (aged 20–28, 7 females, 12 right-eye dominant, 1 author) completed the experiment for a compensation of 50€. The study was run over 2 experimental sessions (on different days) of 12 blocks of approximately 150 minutes each (including breaks). All participants except for 1 author (LW) were naive as to the purpose of the study, and all had normal or corrected to normal vision.

### Setup

Participants sat in a quiet and dimly illuminated room, with their head positioned on a chin and forehead rest. The experiment was controlled by an Apple iMac computer (Cupertino, CA). Manual responses were recorded via a standard keyboard. The dominant eye’s gaze position was recorded and made available online using an EyeLink 1000 Desktop Mount (SR Research, Osgoode, Ontario, Canada) at a sampling rate of 1 kHz. The experimental software controlling the display and the response collection as well as the eye tracking were implemented in Matlab (The MathWorks, Natick, MA), using the Psychophysics [80,81] and EyeLink toolboxes [82]. Stimuli were presented at a viewing distance of 60 cm, on a 24-in Sony GDM F900 CRT screen (Tokyo, Japan) with a spatial resolution of 1,024 × 640 pixels and a vertical refresh rate of 120 Hz [83].

### Experimental design

Each trial began with participants fixating on a central fixation target forming a black (approximately 0 cd/m^2^) and white (approximately 57 cd/m^2^) “bull’s eye” (0.4° radius) on a gray background (approximately 19.5 cd/m^2^). When the participant’s gaze was detected within a 2.0°-radius virtual circle centered on the fixation point for at least 200 ms, the trial began. At that time, 24 distractor streams appeared equally distributed along a 10°-radius imaginary circle centered on the fixation target (see Fig 1A). Distractor streams consisted of flickering stimuli (40 Hz), alternating every 25 ms between a vertical Gabor patch (frequency: 2.5 cpd; 100% contrast; random phase selected each stream refresh; SD of the Gaussian window: 1.1°; mean luminance: approximately 28.5 cd/m^2^) and a Gaussian pixel noise mask (made of approximately 0.22°-width pixels with the same Gaussian envelope as the Gabors). After a random fixation period between 300 and 600 ms (in steps of 1 screen refresh: approximately 8 ms), the fixation target switched off together with the onset of 2 saccade targets. Saccade targets, ST_1_ and ST_2_, were gray circles (approximately 39 cd/m^2^; 1.1° radius; 0.2° width) surrounding 2 randomly chosen streams with an intertarget angular distance of 90° or 30°. They were either presented transiently (50 ms) or continuously (until the end of the trial). When presented transiently, the saccade targets had always disappeared from the screen at the time the discrimination target appeared on the screen. When presented continuously, on the other hand, the saccade targets always temporally overlapped with the presentation of the discrimination target. Our motivation to include these 2 saccade target durations was to check for a potential masking effect of the saccade targets on the discriminability of a discrimination target. Participants were instructed to select 1 of the saccade targets by moving their eyes towards it as fast and as accurately as possible. In 96.5% of all trials, between 75 and 175 ms after the saccade target onset (a time determined to maximize discrimination target offsets in the last 200 ms before the saccade), 1 of the 24 distractor streams was replaced by a discrimination target stream in which a tilted Gabor was played (25 ms, rotated clockwise or counterclockwise by 12° relative to the vertical). The discrimination target could appear at any of the 24 distractor streams with equal probability, and subjects were explicitly informed about this fact at the beginning of the experiment. In 3.5% of all the trials, we did not present any discrimination target, in order to evaluate its influence on saccade metrics (note that all other analyses are based on the discrimination-target-present trials). At 500 ms after the saccade target onset, all stimuli disappeared, and participants were instructed to report the orientation of the discrimination target using the keyboard (right or left arrow key). Incorrect responses were followed by a negative feedback sound. On trials in which no discrimination target was shown, participants’ responses were followed by a random feedback sound.

Three participants were excluded from the analysis because their performance stayed at chance level irrespective of the position of the discrimination target. The remaining 10 participants completed between 6,972 and 7,055 trials of the saccade task. Correct fixation within a 2.0°-radius virtual circle centered on the fixation point was checked online. Trials with fixation breaks were repeated at the end of each block, together with trials during which a saccade started (i.e., crossed the virtual circle around the fixation target) within the first 50 ms or after more than 350 ms following the saccade target onset (participants repeated between 46 to 395 trials across all blocks).

In our experiment, we did not indicate the location of the discrimination target. Therefore, the perceptual task required participants to base their decision on multiple potential locations. One might therefore argue that the low sensitivity at the intermediate location BTW was observed because participants did not take the intermediate location into account as a decision variable for the perceptual task. In order to validate that our results reflect attentional effects and were not selectively biased by varying decision criteria across the different locations, we ran a control experiment, in which the position of the discrimination target was revealed by the presentation of a report cue at the end of each trial. Consequently, participants knew which location to base their discrimination judgment upon in this control experiment, which was—except for the presentation of the report cue—identical to the main experiment. Participants were instructed to give their discrimination judgment only after the report cue had appeared. The report cue (a black circle; approximately 0 cd/m^2^) was presented right after the offset of the distractor streams and stayed on the screen until the trial end. Overall, we tested 8 participants (4 participated in the main experiment) on an equal amount of blocks and trials as in the main experiment. S7 Fig shows the results of this control experiment in the same format as those of the main experiment (see Fig 3).

### Data preprocessing

Before proceeding to the analysis of the behavioral results, we scanned offline the recorded eye-position data. Saccades were detected based on their velocity distribution [84] using a moving average over 20 subsequent eye-position samples. Saccade onset and offset were detected when the velocity exceeded or fell below the median of the moving average by 3 SDs for at least 20 ms. We included trials if a correct fixation was maintained within a 2.0° radius centered on the fixation target, if a correct saccade started at the fixation target and landed at a distance between 7° and 13° from the fixation target (±30% of the instructed saccade size), and if no blink occurred during the trial. Finally, only trials in which the discrimination target offset was included in the last 200 ms preceding the saccade onset were included in the analysis (mean ± SEM discrimination target offset relative to the saccade onset for the selected trials: −50.2 ± 1.3 ms). In total, we included 53,117 trials in the analysis (78.2% of the online-selected trials; 75.7% of all trials played) corresponding to an average of 106.0 ± 2.1 trials (115.9 ± 3.3 no-discrimination-target trials) and 105.3 ± 1.8 trials (125.0 ± 4.4 no-discrimination-target trials) per discrimination target location and participant, in the 90° and 30° conditions, respectively.

Corrective saccades were defined as the saccades directly following the offline-selected main saccades sequence and landing at a distance between 7° and 13° from the fixation target. Corrective saccades were included only if they started before the participant’s behavioral response and within the first 500 ms following the main saccade sequence. In total, we obtained 14,714 corrective saccade trials in the analysis (21.7% of the online-selected trials; 21.0% of all trials played).

### Behavioral data analysis

Before proceeding to any behavioral analysis, we first rotated the trial configuration as to align the 2 saccade target locations (ST1: +45°, ST2: −45° and ST1: +15°, ST2: −15° for the conditions in which they were separated by 90° and 30°, respectively) symmetrically around the geometrical angle 0 (BTW). We then determined the sensitivity to discriminate the orientation of the discrimination targets (d’): d’ = z(hit rate) − z(false alarm rate). To do so, we defined a clockwise response to a clockwise discrimination target (arbitrarily) as a hit and a clockwise response to a counterclockwise discrimination target as a false alarm. Corrected performance of 99% and 1% were substituted if the observed proportion correct was equal to 100% or 0%, respectively. Performance below the chance level (50% or d’ = 0) were transformed to negative d’ values [83]. We analyzed sensitivity as a function of the discrimination position in space irrespective of the saccade landing direction (Fig 3A and 3B) but also as a function of the discrimination target position relative to the saccade landing direction (Fig 3C–3F). To do so, we redefined the position of the discrimination target relative to the saccade direction binned across 24 even, angular sectors of 15° (±7.5° from each distractor stream center angle). This binning was chosen to match with the locations at which we tested visual attention.

We initially computed single-subject means and then averaged these means across participants for each of the compared conditions to get the presented results. For all statistical comparisons, we drew (with replacement) 10,000 bootstrap samples from the original pair of compared values. We then calculated the difference of these bootstrapped samples and derived 2-tailed *p*-values from the distribution of these differences.

Individual raw data and averaged processed data can be found in the Open Science Framework (OSF) online repository at https://osf.io/762up/.

## Supporting information

S1 Fig Saccade direction as a function of saccade latency. (A, B) Plots show the saccade landing direction relative to BTW for all trials as a function of the saccade latency in the 90° (panel A) and 30° (panel B) condition for each participant individually. Dot color indicates the DT location (blue: ST_1_; green: BTW; red: ST_2_; gray: CTRL). Note the overall consistency across participants and DT locations. BTW, position in between the saccade targets; CTRL, control position; DT, discrimination target; ST_1_, saccade target 1; ST_2_, saccade target 2.
(TIF)

S2 Fig Absolute saccade direction and sensitivity as a function of saccade latency. (A, B) Lines show the mean absolute saccade direction relative to BTW grouped into 4 quartiles of saccade latency for the 90° (panel A) and 30° (panel B) condition across all participants irrespective of the discrimination target location. Note that a homogenous distribution of averaging and accurate saccades in the 30° condition should lead to an averaged angle of 10°. (C, D) Mean visual sensitivity (d’), averaged across participants, for trials grouped into 4 quartiles of saccade latency for the 90° (panel C) and 30° (panel D) position. Line color indicates the discrimination target location (blue: ST_1_; green: BTW; red: ST_2_; gray: CTRL). The vertical and horizontal dimensions of the shaded areas around each point represent the SEM. BTW, position in between the saccade targets; CTRL, control position; ST_1_, saccade target 1; ST_2_, saccade target 2.
(TIF)

S3 Fig Absolute saccade direction as a function of saccade latency. (A, B) Lines show the mean absolute saccade direction relative to BTW as a function of the saccade latency grouped into 4 quartiles of saccade latency in the 90° (panel A) and 30° (panel B) condition for each participant individually. Dot color indicates the discrimination target location (blue: ST_1_; green: BTW; red: ST_2_; gray: CTRL). BTW, position in between the saccade targets; CTRL, control position; ST_1_, saccade target 1; ST_2_, saccade target 2.
(TIF)

S4 Fig Sensitivity as a function of saccade latency. (A, B) Lines show sensitivity (d’) as a function of the saccade latency binned into quartiles of trials in the 90° (panel A) and 30° (panel B) condition for each participant individually. Line color indicates the discrimination target location (blue: ST_1_; green: BTW; red: ST_2_; gray: CTRL). BTW, position in between the saccade targets; CTRL, control position; ST_1_, saccade target 1; ST_2_, saccade target 2.
(TIF)

S5 Fig Corrective saccades. (A) Circular plot shows averaged frequency distribution of the corrective saccade landing direction following an averaging saccade. (B) Bar graph illustrates averaged frequency of trials as a function of the corrective saccade landing direction following an averaging saccade for 3 positions of interest (ST_1_, BTW, and ST_2_). (C) Bar graph illustrates sensitivity observed for DT shown at the correctively saccaded (purple) and the correctively nonsaccaded (light purple) positions for trials in which the main saccade was directed in between the saccade target. Conventions are as in Figs 2 and 3. BTW, position in between the saccade targets; DT, discrimination target; ST_1_, saccade target 1; ST_2_, saccade target 2.
(TIF)

S6 Fig Visual sensitivity for different saccade direction grouping procedures in the 30° condition. (A, B) Visual sensitivity (d‘), averaged across participants, as a function of the saccade direction. Data are grouped using ±7.5° (panel A) and ±2.5° (panel B) bins centered on the discrimination target location (in panel B, data are from a running average at each saccade direction degree). Bottom panel shows the amount of trials per data point. Line color indicates the discrimination target location (blue: ST_1_; green: BTW; red: ST_2_). Shaded areas represent the SEM. BTW, position in between the saccade targets; ST_1_, saccade target 1; ST_2_, saccade target 2.
(TIF)

S7 Fig Visual sensitivity in a control experiment, in which the position of the DT was revealed to the participant (*n* = 8; 4 participated in the main experiment) via the presentation of a report cue similar to the saccade targets at the end of each trial. (A, B) Circular plots show averaged visual sensitivity (d’) as a function of the DT position in the 90° (panel A) and 30° (panel B) conditions, irrespective of the duration of the saccade targets and across all saccade directions observed. Bar graphs illustrate visual sensitivity for 4 positions of interest (ST_1_, BTW, ST_2_, CTRL). (C, D) Visual sensitivity as a function of the DT position relative to the saccade landing direction in the 90° (panel C) and 30° (panel D) conditions, irrespective of the duration of the saccade targets (blue: saccade to ST_1_; green: saccade to BTW; red: saccade to ST_2_). For each saccade direction, we took the average sensitivity for each DT location. For example, the blue line plots visual sensitivity when saccades were made towards ST_1_ and the DT was either at ST_1_ (+15° on the polar plot), BTW (15° counterclockwise to ST_1_; 0° on the polar plot), or ST_2_ (30° counterclockwise to ST_1_; +345° on the polar plot), and so on. (E, F) Bar graphs illustrate sensitivity observed for DT shown at the saccaded (purple: e.g., DT at ST_1_ and saccade to ST_1_) and the nonsaccaded (light purple: e.g., DT at ST_1_ and saccade to ST_2_ or BTW) positions in the 90° (panel E) and the 30° (panel F) conditions. Conventions are as in Fig 3. As evident when comparing the results of this control experiment to those of the main experiment (see Fig 3), revealing the location of the DT at the end of the trial did not change the overall pattern of the results. The report cue increased discrimination performance overall but not selectively at any specific location. This control experiment thus demonstrates that the attentional effects reported in the main experiment are immune to potential decision biases. BTW, position in between the saccade targets; CTRL, control position; DT, discrimination target; ST_1_, saccade target 1; ST_2_, saccade target 2.
(TIF)

## Abbreviations

BTW: position in between the saccade targets
CTRL: control position
FEF: Frontal Eye Field
fMRI: functional magnetic resonance imaging
LIP: Lateral Intraparietal Cortex
MT: Middle Temporal Visual Area
SC: Superior Colliculus
ST_1_: saccade target 1
ST_2_: saccade target 2
V: Visual Area
