# Supplementary figures and images for: Visual attention is not deployed at the endpoint of averaging saccades
Source: PLoS Biol. 2018 Jun 25;16(6):e2006548. doi: 10.1371/journal.pbio.2006548 (PMC6034887; doi:10.1371/journal.pbio.2006548)

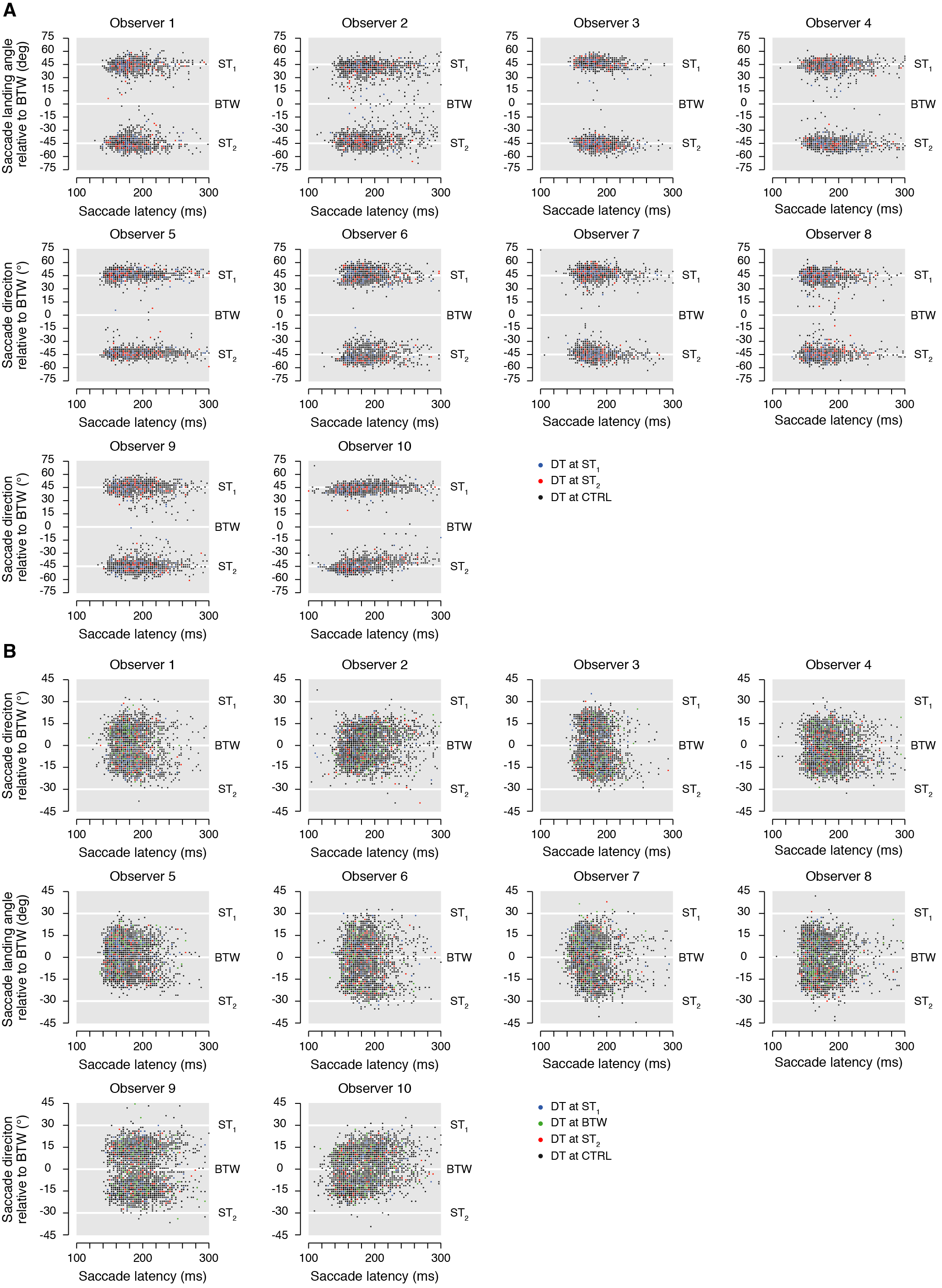

Supplement: S1 Fig — (A, B) Plots show the saccade landing direction relative to BTW for all trials as a function of the saccade latency in the 90° (panel A) and 30° (panel B) condition for each participant individually. Dot color indicates the DT location (blue: ST1; green: BTW; red: ST2; gray: CTRL). Note the overall consistency across participants and DT locations. BTW, position in between the saccade targets; CTRL, control position; DT, discrimination target; ST1, saccade target 1; ST2, saccade target 2. (TIF) [file pbio.2006548.s001.tif]

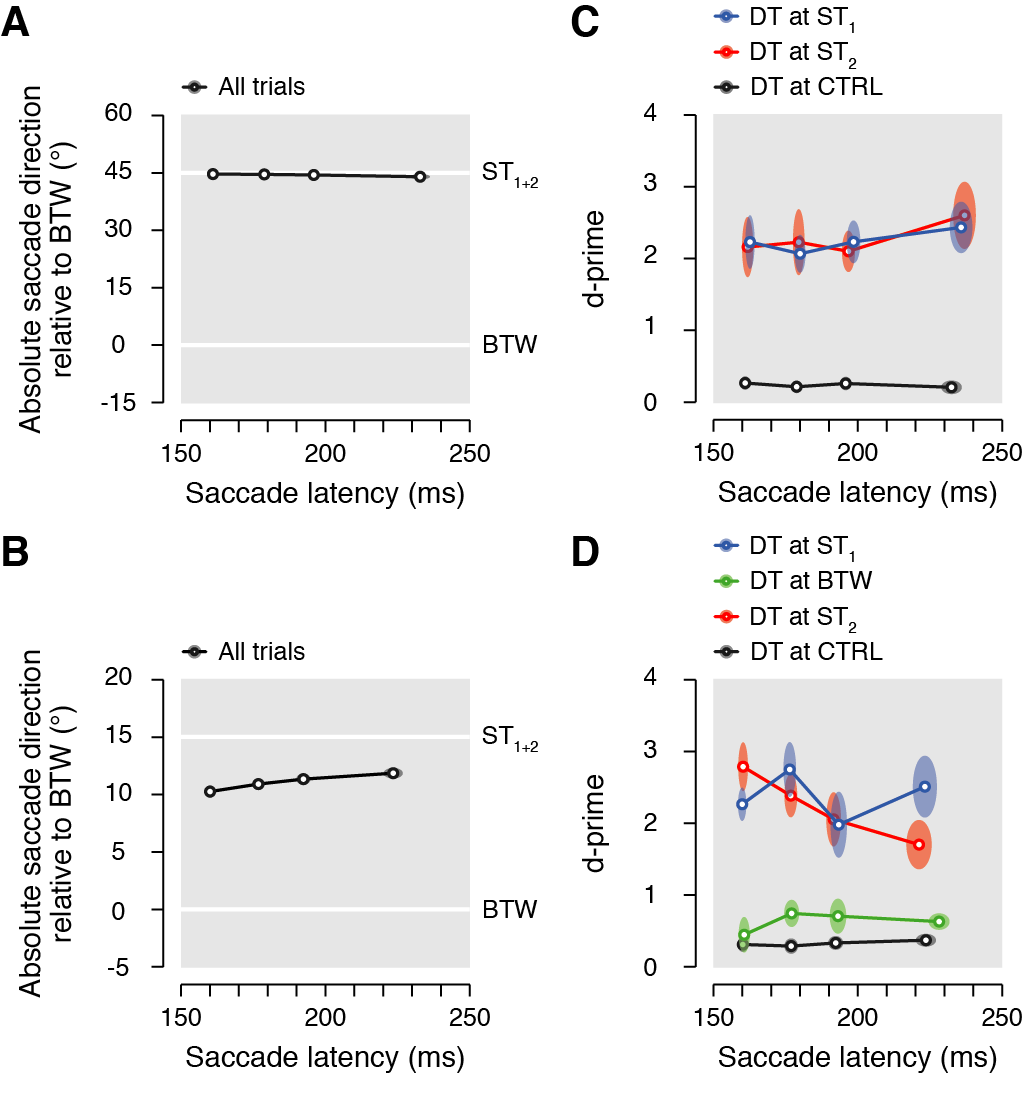

Supplement: S2 Fig — (A, B) Lines show the mean absolute saccade direction relative to BTW grouped into 4 quartiles of saccade latency for the 90° (panel A) and 30° (panel B) condition across all participants irrespective of the discrimination target location. Note that a homogenous distribution of averaging and accurate saccades in the 30° condition should lead to an averaged angle of 10°. (C, D) Mean visual sensitivity (d’), averaged across participants, for trials grouped into 4 quartiles of saccade latency for the 90° (panel C) and 30° (panel D) position. Line color indicates the discrimination target location (blue: ST1; green: BTW; red: ST2; gray: CTRL). The vertical and horizontal dimensions of the shaded areas around each point represent the SEM. BTW, position in between the saccade targets; CTRL, control position; ST1, saccade target 1; ST2, saccade target 2. (TIF) [file pbio.2006548.s002.tif]

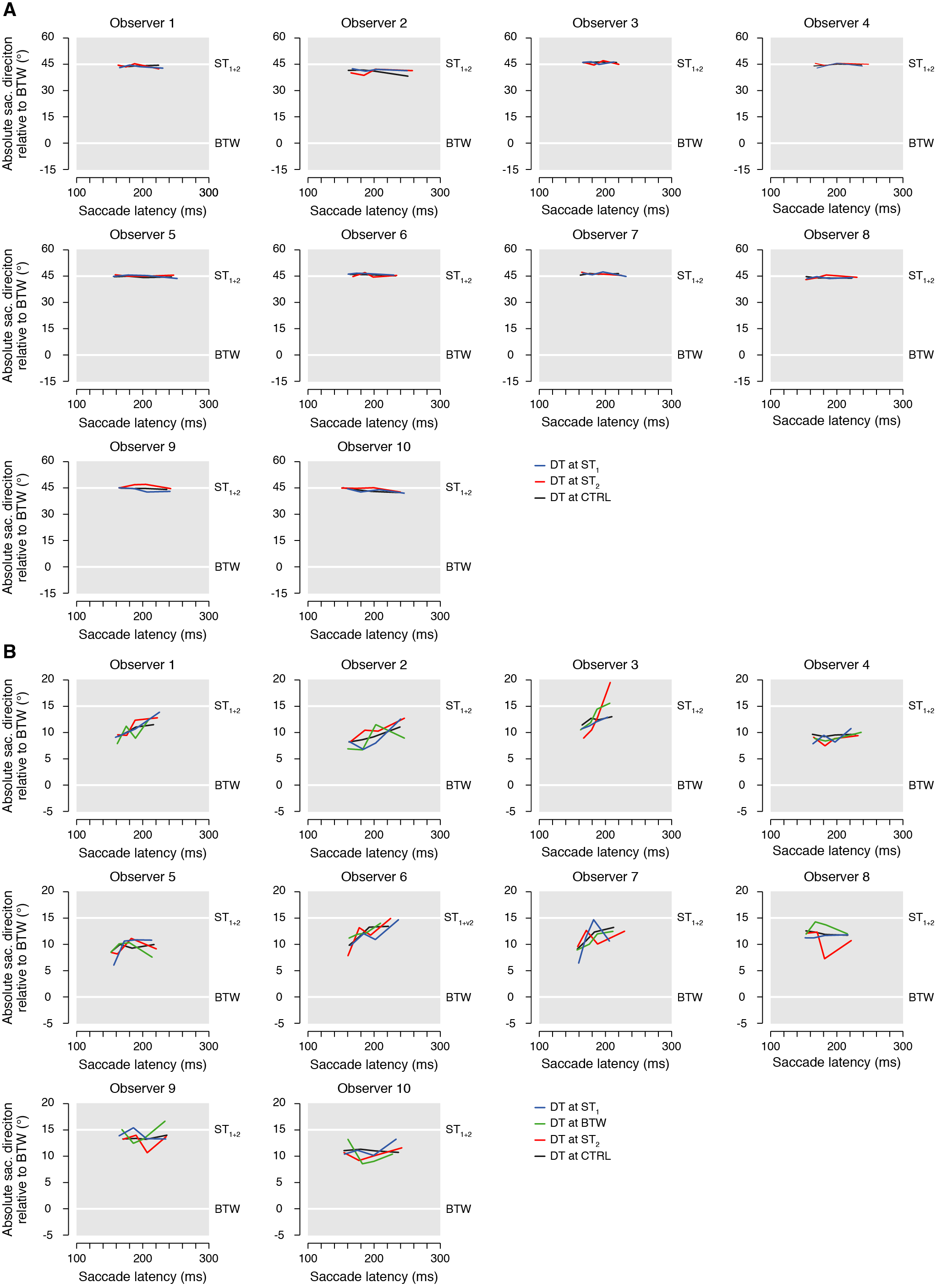

Supplement: S3 Fig — (A, B) Lines show the mean absolute saccade direction relative to BTW as a function of the saccade latency grouped into 4 quartiles of saccade latency in the 90° (panel A) and 30° (panel B) condition for each participant individually. Dot color indicates the discrimination target location (blue: ST1; green: BTW; red: ST2; gray: CTRL). BTW, position in between the saccade targets; CTRL, control position; ST1, saccade target 1; ST2, saccade target 2. (TIF) [file pbio.2006548.s003.tif]

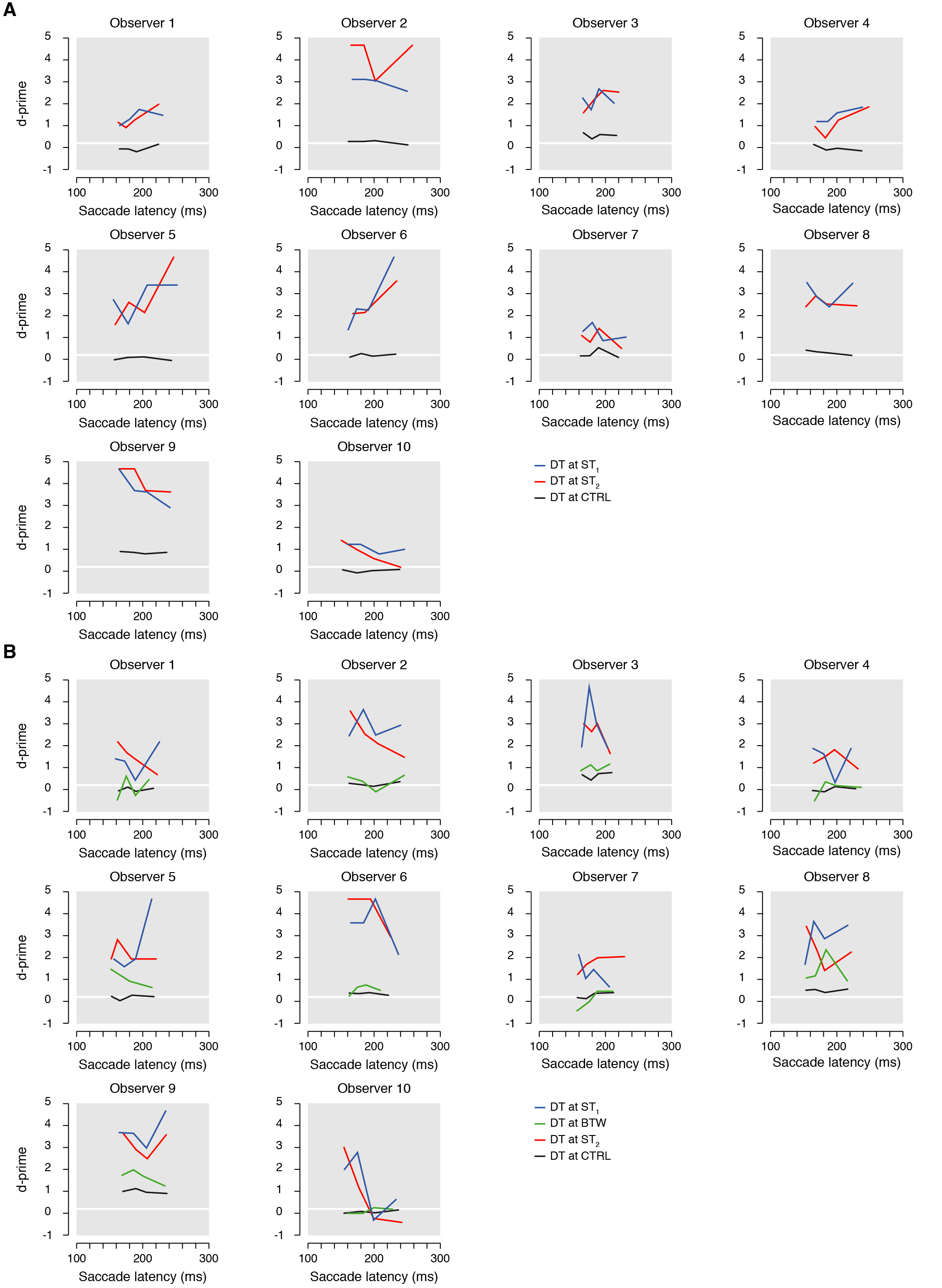

Supplement: S4 Fig — (A, B) Lines show sensitivity (d’) as a function of the saccade latency binned into quartiles of trials in the 90° (panel A) and 30° (panel B) condition for each participant individually. Line color indicates the discrimination target location (blue: ST1; green: BTW; red: ST2; gray: CTRL). BTW, position in between the saccade targets; CTRL, control position; ST1, saccade target 1; ST2, saccade target 2. (TIF) [file pbio.2006548.s004.tif]

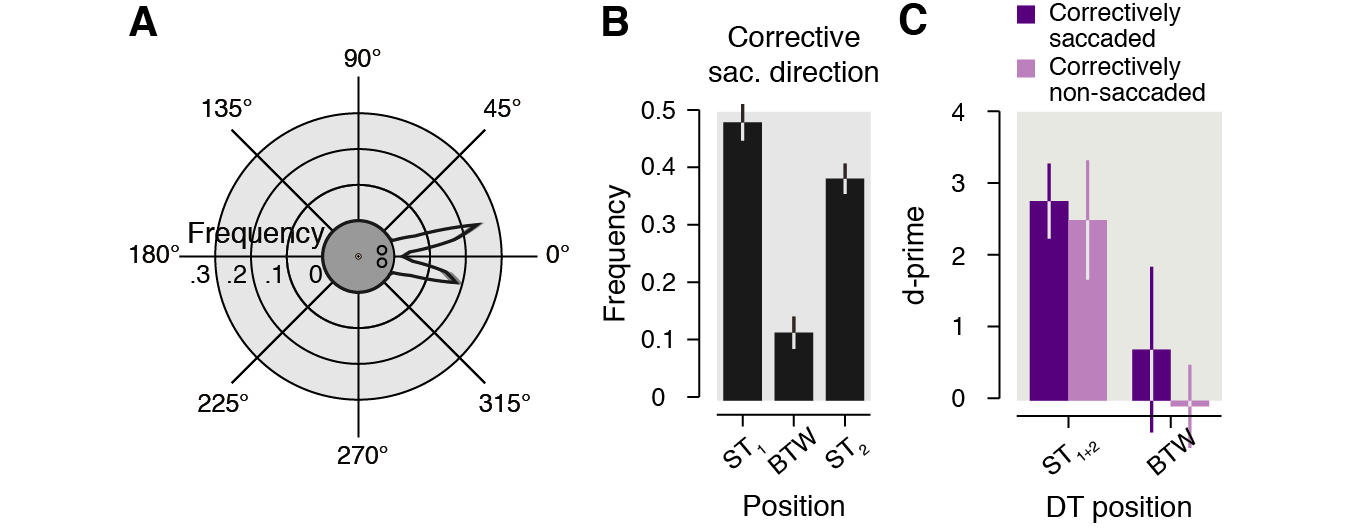

Supplement: S5 Fig — (A) Circular plot shows averaged frequency distribution of the corrective saccade landing direction following an averaging saccade. (B) Bar graph illustrates averaged frequency of trials as a function of the corrective saccade landing direction following an averaging saccade for 3 positions of interest (ST1, BTW, and ST2). (C) Bar graph illustrates sensitivity observed for DT shown at the correctively saccaded (purple) and the correctively nonsaccaded (light purple) positions for trials in which the main saccade was directed in between the saccade target. Conventions are as in Figs 2 and 3. BTW, position in between the saccade targets; DT, discrimination target; ST1, saccade target 1; ST2, saccade target 2. (TIF) [file pbio.2006548.s005.tif]

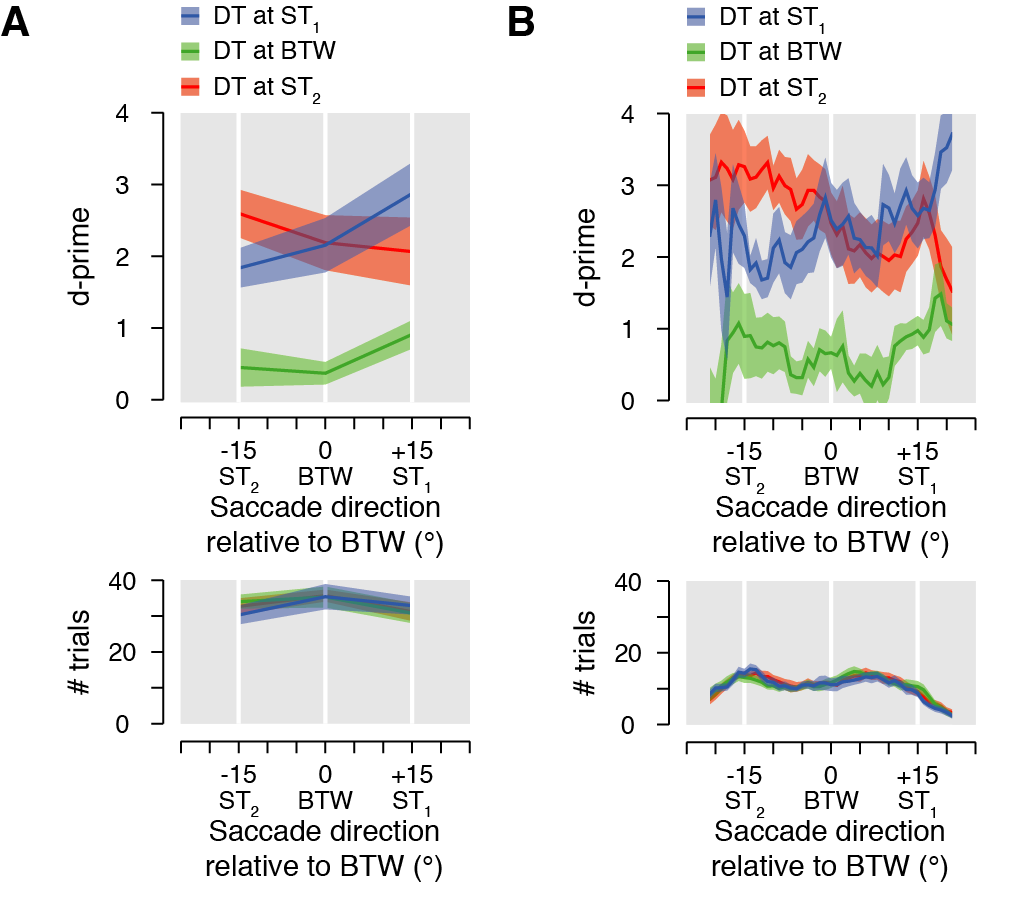

Supplement: S6 Fig — (A, B) Visual sensitivity (d‘), averaged across participants, as a function of the saccade direction. Data are grouped using ±7.5° (panel A) and ±2.5° (panel B) bins centered on the discrimination target location (in panel B, data are from a running average at each saccade direction degree). Bottom panel shows the amount of trials per data point. Line color indicates the discrimination target location (blue: ST1; green: BTW; red: ST2). Shaded areas represent the SEM. BTW, position in between the saccade targets; ST1, saccade target 1; ST2, saccade target 2. (TIF) [file pbio.2006548.s006.tif]

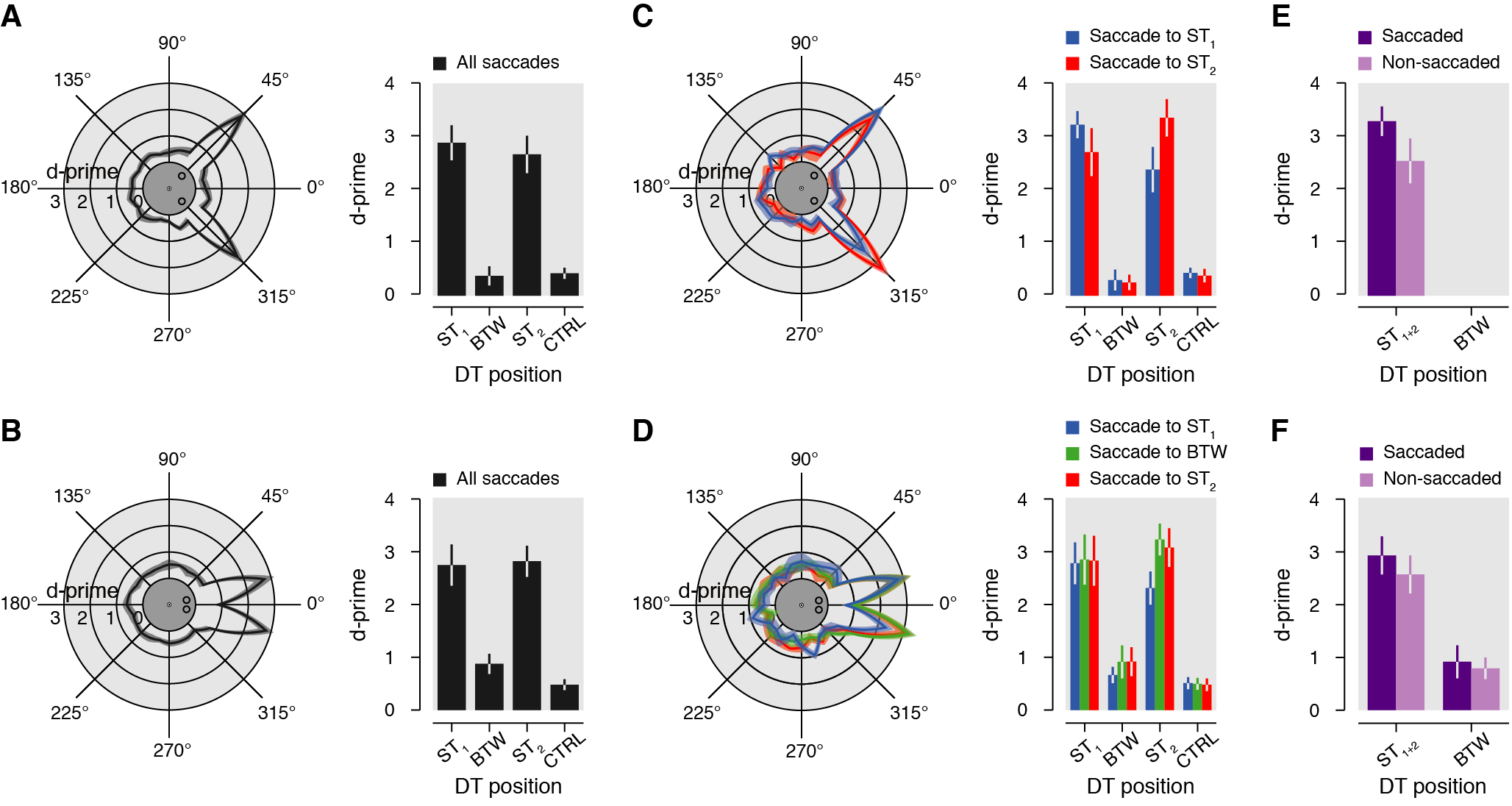

Supplement: S7 Fig — (A, B) Circular plots show averaged visual sensitivity (d’) as a function of the DT position in the 90° (panel A) and 30° (panel B) conditions, irrespective of the duration of the saccade targets and across all saccade directions observed. Bar graphs illustrate visual sensitivity for 4 positions of interest (ST1, BTW, ST2, CTRL). (C, D) Visual sensitivity as a function of the DT position relative to the saccade landing direction in the 90° (panel C) and 30° (panel D) conditions, irrespective of the duration of the saccade targets (blue: saccade to ST1; green: saccade to BTW; red: saccade to ST2). For each saccade direction, we took the average sensitivity for each DT location. For example, the blue line plots visual sensitivity when saccades were made towards ST1 and the DT was either at ST1 (+15° on the polar plot), BTW (15° counterclockwise to ST1; 0° on the polar plot), or ST2 (30° counterclockwise to ST1; +345° on the polar plot), and so on. (E, F) Bar graphs illustrate sensitivity observed for DT shown at the saccaded (purple: e.g., DT at ST1 and saccade to ST1) and the nonsaccaded (light purple: e.g., DT at ST1 and saccade to ST2 or BTW) positions in the 90° (panel E) and the 30° (panel F) conditions. Conventions are as in Fig 3. As evident when comparing the results of this control experiment to those of the main experiment (see Fig 3), revealing the location of the DT at the end of the trial did not change the overall pattern of the results. The report cue increased discrimination performance overall but not selectively at any specific location. This control experiment thus demonstrates that the attentional effects reported in the main experiment are immune to potential decision biases. BTW, position in between the saccade targets; CTRL, control position; DT, discrimination target; ST1, saccade target 1; ST2, saccade target 2. (TIF) [file pbio.2006548.s007.tif]
